# Differences upon admission and in hospital course of children hospitalized with community-acquired pneumonia with or without radiologically-confirmed pneumonia: a retrospective cohort study

**DOI:** 10.1186/s12887-015-0485-6

**Published:** 2015-10-23

**Authors:** Raquel Simbalista, Dafne C. Andrade, Igor C. Borges, Marcelo Araújo, Cristiana M. Nascimento-Carvalho

**Affiliations:** Postgraduate Program in Pathology, Federal University of Bahia School of Medicine, Salvador, Brazil; Postgraduate Program in Health Sciences, Federal University of Bahia School of Medicine, Salvador, Brazil; Image Diagnosis, Image Memorial Unit and Bahia Hospital, Salvador, Brazil; Department of Paediatrics, Federal University of Bahia School of Medicine, Salvador, Brazil

## Abstract

**Background:**

The use of chest radiograph (CXR) for the diagnosis of childhood community-acquired pneumonia (CAP) is controversial. We assessed if children with CAP diagnosed on clinical grounds, with or without radiologically-confirmed pneumonia on admission, evolved differently.

**Methods:**

Children aged ≥ 2 months, hospitalized with CAP diagnosed on clinical grounds, treated with 200,000 IU/Kg/day of aqueous penicillin G for ≥ 48 h and with CXR taken upon admission, without pleural effusion, were included in this retrospective cohort. One researcher, blinded to the radiological diagnosis, collected data on demographics, clinical history and physical examination on admission, daily hospital course during the first 2 days of treatment, and outcome, all from medical charts. Radiological confirmation of pneumonia was based on presence of pulmonary infiltrate detected by a paediatric radiologist who was also blinded to clinical data. Variables were initially compared by bivariate analysis. Multi-variable logistic regression analysis assessed independent association between radiologically-confirmed pneumonia and factors which significantly differed during hospital course in the bivariate analysis. The multi-variable analysis was performed in a model adjusted for age and for the same factor present upon admission.

**Results:**

109 (38.5 %) children had radiologically-confirmed pneumonia, 143 (50.5 %) had normal CXR and 31 (11.0 %) had atelectasis or peribronchial thickening. Children without radiologically-confirmed pneumonia were younger than those with radiologically-confirmed pneumonia (median [IQR]: 14 [7–28 months *versus* 21 [12–44] months; *P* = 0.001). None died. The subgroup with radiologically-confirmed pneumonia presented fever on D1 (33.7 *vs*. 19.1; *P* = 0.015) and on D2 (31.6 % *vs*. 16.2 %; *P* = 0.004) more frequently. The subgroup without radiologically-confirmed pneumonia had chest indrawing on D1 (22.4 % *vs*. 11.9 %; *P* = 0.027) more often detected. By multi-variable analysis, Fever on D2 (OR [95 % CI]: 2.16 [1.15-4.06]) was directly and independently associated with radiologically-confirmed pneumonia upon admission.

**Conclusion:**

The compared subgroups evolved differently.

## Background

Community acquired pneumonia (CAP) is the leading cause of mortality in children aged less than 5 years, accounting for 1.1 million childhood deaths every year – more than AIDS, measles and malaria all together [1]. Considering CAP control a fundamental step to achieve the Millennium Development Goal 4 of “reducing by two-thirds, between 1990 and 2015, the under-five mortality rate” [2], the World Health Organization (WHO) proposed in 1990 a standardized case-management protocol for CAP, based solely on symptoms and signs [3]. In 2005, a standardized manual for pneumonia recognition on chest radiograph (CXR) was also produced specifically for epidemiological studies [4].

However, the use of CXR in the lack of a simple gold-standard exam for pneumonia has been questioned in the literature as a practice able to improve clinical outcome [5]. So far, the evidence suggests that an admission CXR has no effect on the outcome of paediatric outpatients with CAP [6]. The inability to distinguish between viral and bacterial aetiology in CAP represents another limitation of CXR analyses [7]. The interpretation of CXR may also be difficult in young children, when a poor inter-observer concordance between attending physicians at the emergency room is demonstrated [8]. Considering the aforementioned aspects of CXR, the British Thoracic Society recommended that CXR should not be considered a routine investigation in children thought to have CAP [9].

Of note, the Pediatric Infectious Diseases Society and the Infectious Diseases Society of America’s guidelines state that CXR (postero-anterior and lateral views) should be obtained in all children hospitalized for management of CAP [10]. It is important to realize that a significant proportion of paediatric CAP cases diagnosed on clinical grounds actually have a normal CXR. For example, in Pakistan, 82 % of the children aged 2–59 months with CAP diagnosed according to the WHO criteria had a normal CXR [11]. To the best of our knowledge, the differences in progression of symptoms and signs between children with CAP diagnosed on clinical grounds with or without radiological confirmation has been assessed only once. That study included 382 children with non-severe CAP, and demonstrated earlier resolution of the symptoms in children with normal CXR. It was also reported that persistence of symptoms such as fever and tachypnoea was predictive of radiologically-confirmed pneumonia [12].

The use of aqueous penicillin G is the recommended antibiotic therapy for all children with CAP who require hospitalization [10]. The rationale for this approach is to treat the bacterial CAP cases caused by *Streptococcus pneumoniae*, which is the most frequent aetiological agent of CAP [13]. Moreover, aqueous penicillin G has treated successfully a massive majority of children hospitalized with CAP [14].

In this context, the aim of this study was to assess if there were differences in hospital course and in outcome between groups of children hospitalized with CAP, diagnosed on clinical grounds, treated with aqueous penicillin G, with or without radiologically-confirmed pneumonia on admission.

## Methods

This retrospective cohort included children aged ≥ 2 months hospitalized with CAP and treated intravenously with 200,000 IU/Kg/day of aqueous penicillin G for at least 48 h, and with CXR taken on admission, in a 37-month period (from October 2002 to October 2005), at the Federal University of Bahia Hospital, in Salvador, North-eastern Brazil. The exclusion criteria comprised underlying debilitating conditions such as heart disease with hemodynamic repercussion, chronic lung disease except asthma, severe malnutrition, immunodeficiency, nosocomial pneumonia from another hospital, transfers to other hospitals during aqueous penicillin G treatment, presence of pleural effusion upon admission and radiological diagnoses other than pneumonia or normal CXR or atelectasis or peribronchial thickening. In accordance with the recommendation from the Brazilian Society of Paediatrics, aqueous penicillin G was the standardized treatment for all children hospitalized with a clinical diagnosis of CAP [15]. Sample size was estimated considering a smaller expected frequency of 15 % and an expected difference between the compared frequencies of 10 %. The sample size was thus estimated as 250 cases in the study group, considering a significance level of 0.05 (95 Confidence Interval [95 %CI]) and power of 80 %.

Based on the hospital admittance log-book, which contained the list of all hospitalized children and the respective cause of hospitalization, one researcher (RS) identified all children hospitalized with CAP during the study period and collected data from the medical charts whilst being blinded to the radiological diagnosis. A paediatric radiologist (MA) blinded to clinical data read the CXR taken on admission and registered the findings in a standardized form for the purpose of this study. He looked for the presence of pulmonary infiltrate, pleural effusion, atelectasis, hyperinflation, abscess, peribronchial thickening, pneumatocele and pneumothorax, taking into account previously published definitions [4]. The final radiological confirmation of pneumonia was based on the presence of pulmonary infiltrate [4].

Data on demographics, clinical history, physical examination on admission, treatment, daily hospital course during the first 2 days of treatment (cough, breathlessness, axillary temperature, respiratory rate, cyanosis, chest indrawing, chest retraction, somnolence, nasal flaring, grunting, seizure), and outcome were collected from the medical charts and recorded on a pre-defined form. For axillary temperature and respiratory rate (RR), the highest registered grade was collected. Fever was defined as axillary temperature ≥ 37.5 °C [16] and tachypnoea as RR ≥ 50 breaths/min in children aged 2–11 months, RR ≥ 40 breaths/min in children from 12 to 59 months of age [17], and RR ≥ 30 in children aged ≥ 60 months [18]. Nutritional evaluation was performed using the software Anthro, version 1.02 (CDC [Center for Disease Control and Prevention] and WHO) and malnutrition and severe malnutrition were defined as Z-score for weight-for-age index under −2.00 or −3.00, respectively, using the National Centre for Health Statistics standard [19].

CAP was classified as non-severe, severe or very severe according to WHO guidelines: patients with chest indrawing were classified as severe CAP and patients with somnolence, seizures, grunting when calm, nasal flaring, cyanosis, or inability to drink were classified as very severe CAP [17]. If a child had chest indrawing along with any item that would classify him/her as very severe CAP, the final classification was very severe CAP.

We compared the frequency of demographic and clinical findings detected upon admission and on each day of hospital course up to the 2^nd^ day between patients with radiologically-confirmed pneumonia and those with normal CXR or without radiologically-confirmed pneumonia. This last group comprised patients with normal CXR or CXR with atelectasis or peribronchial thickening. A subgroup comparison was performed when wheezers were excluded. We also compared the frequency of length of hospital stay and treatment as well as the final outcome upon discharge between these groups. Categorical variables were compared by using chi-square or Fisher exact test as appropriate, and continuous variables were assessed by using Mann–Whitney U test due to non-parametrical distribution. Multi-variable logistic regression analysis by enter method was used to assess independent association between radiologically-confirmed pneumonia and factors which significantly differed during hospital course in the bivariate analysis. The multi-variable analysis was performed in a model adjusted for age and for the same factor present upon admission. The statistical tests were two tailed, with a significance level of 0.05. The software SPSS (version 9.0, IBM, Armonk, New York) was used for the analysis. The exclusion criteria were chosen for the purpose of addressing potential confounders. Blinding to the radiological diagnosis during medical charts review was performed to address potential bias.

The study was conducted according to the principles expressed in the Declaration of Helsinki and it was approved by the Ethics Committee at Federal University of Bahia. Informed consent was deemed unnecessary due to the retrospective collection of data. Identification of the patients was kept confidential.

## Results

**Fig. 1:**
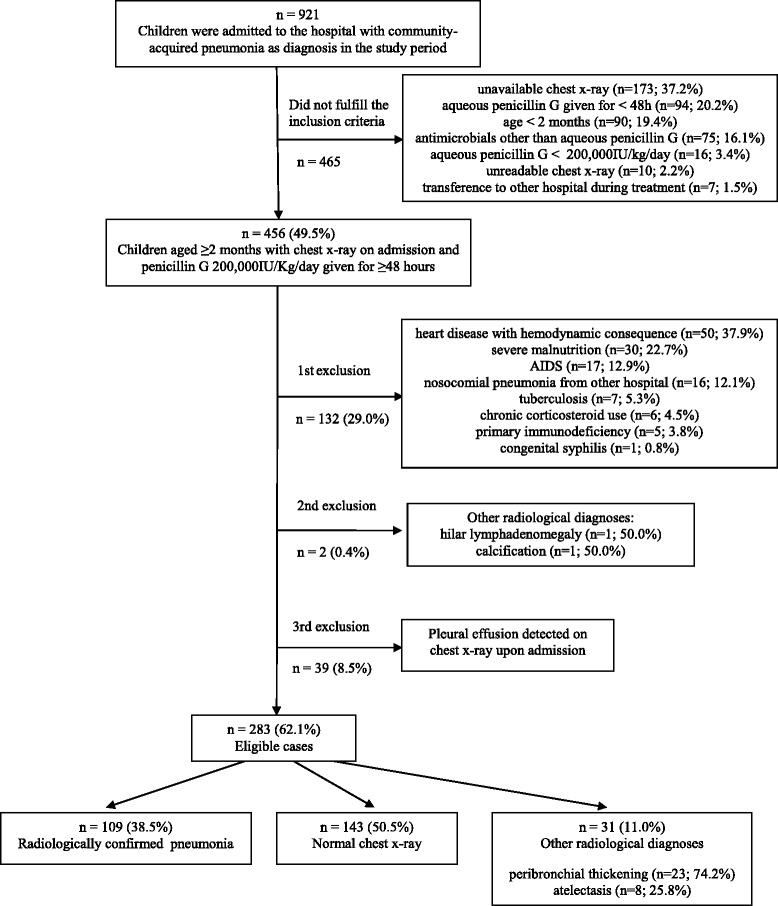
Flow-chart of the step-by-step selection of children hospitalized with community-acquired pneumonia diagnosed on clinical grounds

During the study period, 921 cases were detected and 456 patients fulfilled the inclusion criteria. After excluding 132 (29.0 %) cases due to underlying debilitating illnesses, a further 39 (8.5 %) with pleural effusion detected on the CXR taken upon admission, and an additional 2 (0.4 %) due to other radiological diagnoses such as calcification and hilar lymphadenomegaly (Fig. 1), the final study group comprised 283 (62.1 %) patients. Overall, 157 (55.5 %) patients were males, the median age was 17 months (IQR [interquartile range]: 9–34 months; minimum 2 months; maximum 9.2 years) and 101 (35.7 %) patients were aged under 1 year. Upon admission, the most common complaints were cough (86.2 %), fever (84.8 %), breathlessness (67.5 %), and the most frequent findings were tachypnoea (76.9 %), fever (53.0 %), crackles (50.2 %), wheezing (46.3 %), chest retraction (37.8 % ) and chest indrawing (34.3 %). CAP was severe or very severe among 77 (27.2 %) and 33 (11.7 %) patients, respectively. Malnutrition was detected in 21 (7.4) cases and severe malnutrition in 1 (0.4 %) case.

The compared subgroups included 109 (38.5 %) children with radiologically-confirmed pneumonia, 143 (50.5 %) children with normal CXR and 31 (11.0 %) with other radiological diagnoses (atelectasis or peribronchial thickening). In the radiologically-confirmed pneumonia subgroup, pulmonary infiltrate was classified as alveolar (94.5 %), alveolar-interstitial (3.7 %) or interstitial (1.8 %). Additional radiological findings were atelectasis (2.8 %) and peribronchial thickening (3.7 %). The baseline characteristics are compared in Table 1. Children without radiologically-confirmed pneumonia were younger than those with radiologically-confirmed pneumonia (median [IQR]: 14 [7–28 months *versus* 21 [12–44] months; *P* = 0.001). No difference was found in the frequency of malnutrition (10 [9.2 %] *versus* 11 [6.3 %]; *P* = 0.373).

**Table 1:** Baseline and clinical characteristics of children hospitalized with community-acquired pneumonia diagnosed on clinical grounds

Characteristics	Radiologically-confirmed pneumonia				
	Yes (*n* = 109)	Normal CXR (*n* = 143)	*P*	No^e^ (*n* = 174)	*P*
Gender male^a^	70 (64.2)	77 (53.8)	0.098	87 (50.0)	0.019
Age strata^a, b^					
2-11 months	28 (25.7)	63 (44.1)	0.003	73 (42.0)	0.005
1-4 years	67 (61.5)	68 (47.6)	0.028	87 (50.0)	0.059
≥ 5 years	14 (12.8)	12 (8.4)	0.250	14 (8.0)	0.188
History of current illness					
fever^a^	100 (91.7)	112 (78.3)	0.004	140 (80.5)	0.010
duration of fever^c^	*n* = 79	*n* = 80		*n* = 105	
	5 (3–7); 1-30	4(2–6); 1-20	0.093	4(2–7);1-20	0.299
cough^a^	92 (84.4)	123 (86.0)	0.720	152 (87.4)	0.483
duration of cough^c^	*n* = 59	*n* = 80		*n* = 93	
	7 (4–9); 1-45	4.5 (3–7); 1-31	0.022	5(3–7.5);1-31	0.053
breathlessness^a^	67 (61.5)	104 (72.7)	0.058	124 (71.3)	0.087
duration of breathlessness^c^	*n* = 46	*n* = 77		*n* = 93	
	2 (1–6); 1-30	3 (1–4.5); 1-30	0.894	3(1–5);1-30	0.504
Physical examination findings					
tachypnoea^a^	68/85^d^ (80.0)	79/106^d^ (74.5)	0.372	98/131^d^ (74.8)	0.377
fever^a^	52/99^d^ (52.5)	62/121^d^ (51.2)	0.849	79/148^d^ (53.4)	0.895
crackles^a^	42 (38.5)	86 (60.1)	0.001	100 (57.5)	0.002
wheezing^a^	32 (29.4)	86 (60.1)	<0.001	99 (56.9)	<0.001
chest retraction^a^	38 (34.9)	53 (37.1)	0.719	69 (39.7)	0.418
Severity according to WHO^b^					
non-severe^a^	76 (69.7)	76 (53.1)	0.008	97 (55.7)	0.019
severe^a^	22 (20.2)	47 (32.9)	0.025	55 (31.6)	0.036
very severe^a^	11 (10.1)	20 (14.0)	0.351	22 (12.6)	0.515
chest indrawing^a^	30 (27.5)	58 (40.6)	0.032	67 (38.5)	0.058
nasal flaring^a^	7 (6.4)	17 (11.9)	0.143	19 (10.9)	0.202
somnolence^a^	1 (0.9)	1 (0.7)	1.000	1 (0.6)	1.000
seizure^a^	1 (0.9)	1 (0.7)	1.000	1 (0.6)	1.000
cyanosis^a^	2 (1.8)	1 (0.7)	0.580	1 (0.6)	0.561

CXR indicates chest radiograph

WHO indicates World Health Organization

^a^Data are shown as n (%)

^b^The frequencies in each age stratum or in the severity groups according to WHO were compared as dichotomic variables

^c^Data are shown as median (IQR); minimum-maximum

^d^Different denominators are due to missing data

^e^Includes normal CXR plus CXR with atelectasis or peribronchial thickening

Overall, the median duration of hospitalization was 7 days (IQR: 5–10; minimum 2; maximum 31), and the median duration of aqueous penicillin G use was 4 days (IQR: 3–6; minimum 2; maximum 17). Children with radiologically-confirmed pneumonia stayed in hospital for as long as children without radiologically-confirmed pneumonia (median 7 days [IQR: 4–10] *versus* median 7 days [IQR: 5–9]; *P* = 0.903). No difference was found between the two subgroups regarding duration of penicillin use (radiologically-confirmed pneumonia: median 4 days [IQR: 3–6] *versus* no radiologically-confirmed pneumonia: median 4 days [IQR: 3–6]; *P* = 0.402). Overall, aqueous penicillin G was substituted by other antibiotics in 29 (10.2 %) cases. Children with radiologically-confirmed pneumonia had aqueous penicillin G substituted more frequently than those without radiologically-confirmed pneumonia (15.6 % *versus* 6.9 %; *P* = 0.019).

No patient died and everyone was discharged after improvement. Table 2 presents the significant differences found during progression of disease between children with or without radiologically-confirmed pneumonia or normal CXR during aqueous penicillin G treatment. Those with substitution of aqueous penicillin G were excluded. The comparison of the symptoms and signs during hospital course which did not demonstrate significant difference is shown in Table 3. Table 4 depicts the multi-variable analysis of factors whose difference was significant in the bivariate analysis presented in Table 2.

**Table 2:** Significant differences during hospital course of children hospitalized with community-acquired pneumonia diagnosed on clinical grounds

Characteristics	Radiologically-confirmed pneumonia				
	Yes	Normal CXR	*P*	No^d^	*P*
D1^a^	*n* = 109^b^	*n* = 143^b^		*n* = 174	
Fever	29/86^c^ (33.7)	17/107^c^ (15.9)	0.004	25/131 (19.1)	0.015
Chest indrawing	13 (11.9)	31 (21.7)	0.043	39 (22.4)	0.027
D2^a^	*n* = 109^b^	*n* = 143^b^			
Fever	31/98^c^ (31.6)	16/119^c^ (13.4)	0.001	24/148 (16.2)	0.004
Without wheezers					
D1^a^					
Fever	26/62 (41.9)	9/46 (19.6)	0.014	14/59 (23.7)	0.033
Chest indrawing	6/77 (7.8)	9/57 (15.8)	0.147	12/75 (16.0)	0.117
D2^a^					
Fever	28/69 (40.6)	10/50 (20.0)	0.017	15/68 (22.1)	0.020

Data are shown as n (%)

CXR indicates chest radiograph

^a^ *D1* is the first day after aqueous penicillin G has been initiated (24 h of treatment), *D2* is the second day after aqueous penicillin G has been initiated (48 h of treatment)

^b^ *n* = number of evaluated patients in each subgroup on the respective day of hospital course

^c^Different denominators due to missing data

^d^Includes normal CXR plus CXR with atelectasis or peribronchial thickening

**Table 3:** Symptoms and signs without significant differences during hospital course of children hospitalized with community-acquired pneumonia diagnosed on clinical grounds

Characteristics	Radiologically-confirmed pneumonia				
	Yes	Normal CXR	*P*	No^d^	*P*
D1^a^	*n* = 109^b^	*n* = 143^b^		*n* = 174^b^	
Tachypnoea	54/90^c^ (60.0)	51/107^c^ (47.7)	0.084	65/132^c^ (49.2)	0.115
Cyanosis	0	0	-	0	-
Chest retraction	21 (19.3)	29 (20.3)	0.842	37 (21.3)	0.685
Somnolence	1 (0.9)	1 (0.7)	1.000	1 (0.6)	1.000
Nasal flaring	4 (3.7)	4 (2.8)	0.730	5 (2.9)	0.737
Grunting	0	1 (0.7)	1.000	1 (0.6)	1.000
Seizure	0	0	-	0	-
Cough	41 (37.6)	44 (30.8)	0.255	55 (31.6)	0.299
Breathlessness	18 (16.5)	33 (23.1)	0.199	42 (24.1)	0.127
D2^a^	*n =* 109^b^	*n* = 143^b^		*n =* 174^b^	
Tachypnoea	45/93^c^ (48.4)	52/119^c^ (43.7)	0.496	67/145^c^ (46.2)	0.742
Cyanosis	1 (0.9)	0	0.433	0	0.385
Chest indrawing	11 (10.1)	14 (9.8)	0.937	19 (10.9)	0.826
Chest retraction	14 (12.8)	16 (11.2)	0.688	20 (11.5)	0.734
Somnolence	2 (1.8)	0	0.186	0	0.148
Nasal flaring	1 (0.9)	1 (0.7)	1.000	1 (0.6)	1.000
Grunting	0	1 (0.7)	1.000	1 (0.6)	1.000
Seizure	0	0	-	0	-
Cough	44 (40.4)	65 (45.5)	0.419	79 (45.4)	0.406
Breathlessness	25 (22.9)	28 (19.6)	0.517	34 (19.5)	0.494

Data are shown as n (%)

CXR indicates chest radiograph

^a^ *D1* is the first day after aqueous penicillin G has been initiated (24 h of treatment), *D2* is the second day after aqueous penicillin G has been initiated (48 h of treatment)

^b^ *n* = number of evaluated patients in each subgroup on the respective day of hospital course

^c^Different denominators due to missing data

^d^Includes normal CXR plus CXR with atelectasis or peribronchial thickening

**Table 4:** Multi-variable analysis of factors associated with radiologically-confirmed pneumonia during hospital course in bivariate analysis, adjusted for age and for the same factor upon admission, among children hospitalized with community-acquired pneumonia diagnosed on clinical grounds

	Compared subgroup					
	Normal CXR		CXR without pneumonia		Without wheezers	
Characteristics	OR (95 % CI)	*P*	OR (95 % CI)	*P*	OR (95 % CI)	*P*
Fever on D1^a^	2.18 (1.07-4.43)	0.031	1.75 (0.92-3.34)	0.091	2.00 (0.89-4.48)	0.094
Age	1.00 (1.00-1.00)	0.062	1.00 (1.00-1.00)	0.023	1.00 (0.99-1.00)	0.089
Report of fever upon admission	4.01 (1.54-10.42)	0.004	3.47 (1.35-8.94)	0.010	1.75 (0.58-5.23)	0.317
Chest indrawing on D1^a^	0.65 (0.31-1.37))	0.259	0.60 (0.29-1.22)	0.160	0.60 (0.20-1.77)	0.354
Age	1.00 (1.00-1.00)	0.033	1.00 (1.00-1.00)	0.024	1.00 (1.00-1.00)	0.072
Chest indrawing upon admission	0.67 (0.38-1.19)	0.174	0.74 (0.43-1.28)	0.281	0.67 (0.30-1.46)	0.311
Fever on D2^a^	2.66 (1.32-5.36)	0.006	2.16 (1.15-4.06)	0.016	2.24 (1.04-4.79)	0.039
Age	1.00 (1.00-1.00)	0.086	1.00 (1.00-1.00)	0.044	1.00 (0.99-1.00)	0.116
Report of fever upon admission	4.15 (1.61-10.67)	0.003	3.65 (1.44-9.23)	0.006	2.01 (0.69-5.83)	0.199

Multi-variable analysis by logistic regression

CXR indicates chest radiograph

CXR without pneumonia includes normal CXR plus CXR with atelectasis or peribronchial thickening

^a^ *D1* is the first day after aqueous penicillin G has been initiated (24 h of treatment), *D2* is the second day after aqueous penicillin G has been initiated (48 h of treatment)

## Discussion

This study provides evidence that children hospitalized with CAP diagnosed on clinical grounds treated with aqueous penicillin G, present differences during hospital course when radiologically-confirmed pneumonia cases are compared to others without radiologically-confirmed pneumonia or with normal CXR. Notably, patients with radiologically-confirmed pneumonia were significantly more feverish on admission and during the first 2 days of aqueous penicillin G use. This finding remained when wheezers were excluded from the analysis. It is important to recall that children included in this study were otherwise healthy and had no significant comorbidity.

Several methodological constraints should be highlighted in this investigation. As data were collected retrospectively, there was no control on variables measurement and, as patients were evaluated by different observers, standardization of evaluations could not be guaranteed. Also, no aetiological agent was determined. However, strict criteria for enrolling and grouping the cases were used, and those with potential confounding variables were excluded. Moreover, the study was performed in a teaching hospital where the same standardized procedures for assistance have been used over the period of the study [15]. Interestingly, all children included in the analysis had pneumonia diagnosed and were admitted to hospital by paediatricians.

The presence of fever has been lately associated with radiologically-confirmed pneumonia. A recent study has estimated that presence of fever increases the chance of children hospitalized with lower respiratory tract disease to have radiologically-confirmed pneumonia by 2.5 times [20]. Additionally, it has been demonstrated that the inclusion of fever in the WHO criteria for the clinical diagnosis of CAP substantially increases its specificity, particularly in children with wheezing [21]. The history of fever has also been recognized as the symptom with the greatest sensitivity for the presence of pulmonary infiltrates [22]. Our data provide evidence that persistence of fever up to the second day of treatment is also more frequent among hospitalized children with radiologically-confirmed pneumonia.

In a previous investigation which compared the progression of symptoms among children with non-severe acute lower respiratory tract infection with and without a radiological diagnosis of pneumonia, tachypnoea persisted longer during treatment among those with radiologically-confirmed pneumonia [12]. Herein, this finding was not found, possibly due to sample size. Children without radiologically-confirmed pneumonia had higher frequency of wheezing, which is a potential confounding factor for the diagnosis of CAP among children with tachypnoea [23, 24]. The high frequency of children with a clinical diagnosis of CAP and without radiologically-confirmed pneumonia is in accordance with previous studies. Up to 82 % of children with tachypnoea and wheezing had normal CXR in Pakistan [11]. The prescription of antibiotics based on only tachypnoea should be restricted to settings where CXR performance is not feasible. The lower frequency of fever [23] and the younger age [25] in the subgroup without radiologically-confirmed pneumonia may also guide the clinical suspicion to lower respiratory tract diseases other than CAP, for example bronchiolitis.

The evidence that there is no effect of an admission CXR in the outcome of paediatric outpatients with CAP was provided in a study in which all those children, irrespective of having CXR taken, received antibiotics. That means, those who needed antibiotics received antibiotics, as well as those who did not need antibiotics but instead had a self-limited disease [6]. It has been recently shown that radiologically-confirmed pneumonia is associated with bacterial infection [26]. Although CXR is undoubtedly limited in determining the aetiology of pneumonia [7], it may help identify children with a lower respiratory tract disease and a probable non-bacterial aetiology, such as bronchiolitis, who can benefit from not receiving unnecessary antibiotics.

## Conclusions

This is the first study to demonstrate the differences in hospital course between hospitalized children with CAP diagnosed on clinical grounds with or without radiologically-confirmed pneumonia. We highlight differences on the hospital course between the studied subgroups. The performance of CXR may be a tool to select patients who would not benefit from receiving antibiotics and could be followed-up instead.
